# High prevalence of hepatitis delta virus in Cameroon

**DOI:** 10.1038/s41598-018-30078-5

**Published:** 2018-08-02

**Authors:** Emily K. Butler, Mary A. Rodgers, Kelly E. Coller, Devin Barnaby, Elizabeth Krilich, Ana Olivo, Michael Cassidy, Dora Mbanya, Lazare Kaptue, Nicaise Ndembi, Gavin Cloherty

**Affiliations:** 1 Infectious Disease Research, Abbott Laboratories, Abbott Park, IL USA; 2 Franciscan Institute for Science and Health, Franciscan University of Steubenville, Steubenville, OH USA; 3 Department of Hematology, Université de Yaoundé I, Yaoundé, Cameroon; 4 Laboratoire D’Hematologie, Université des Montagnes, Bangangté, Cameroon; 5 Laboratory Research Department, Institute of Human Virology, Abuja, Nigeria

## Abstract

Hepatitis delta virus (HDV), a satellite virus of hepatitis B virus (HBV), infects an estimated 15–20 million people worldwide and confers a greater risk for accelerated progression to liver disease. However, limited HDV surveillance data are available in sub-Saharan Africa where HDV diversity is high. To determine the prevalence and diversity of HDV in Cameroon, serological and molecular characterization was performed on 1928 HBsAg positive specimens selected from retrospective viral surveillance studies conducted in Cameroon from 2010–2016. Samples were screened for HDV antibodies on the Abbott ARCHITECT instrument and for HDV RNA on the Abbott m2000 instrument by research assays. HDV positive specimens with sufficient viral load were selected for genomic sequencing. The seroprevalence of HDV in HBsAg positive samples from Cameroon was 46.73% [95% CI; 44.51–48.96%], with prevalence of active HDV infection being 34.2% [95% CI; 32.09–36.41%]. HDV genotypes 1, 6, 7 and 8 were identified amongst N = 211 sequences, including N = 145 genomes. HDV prevalence is high within the study cohort, indicating that a large portion of HBV infected individuals in Cameroon are at elevated risk for severe hepatitis and death. Collectively, these results emphasize the need for HBV vaccination and HDV testing in HBsAg positive patients in Cameroon.

## Introduction

Hepatitis delta virus (HDV) is the smallest virus known to infect humans. HDV infection is restricted to co-infection with hepatitis B virus (HBV) or superinfection of HBV-infected individuals due to the requirement for HBV surface antigen (HBsAg) in the HDV viral envelope. An estimated 15–20 million people are co-infected with HBV and HDV worldwide; with areas of high endemicity in the Middle East, the Amazonian region of South America, the Mediterranean, and western and central Africa^1^. The impact of HDV infection is substantial: co-infection results in a more severe clinical outcome than HBV alone, while superinfection of HBV infected patients with HDV results in greater probability of chronic infection, more rapid progression to cirrhosis^2^, and overall greater risk of morbidity^3^. Further, hepatitis treatment regimens which decrease HBV viral loads (e.g. nucleos(t)ide analogs) do not impact HDV and pegylated interferon alpha shows poor efficacy with relapse common^4,5^. Additionally, HDV inhibits HBV replication, lowering HBV DNA levels detectable in patient blood^6–8^, and thereby potentially complicating HBV patient management. The ability to detect and monitor for HDV is clearly critical to patient care and the need for novel therapeutics is imperative^9–12^, especially in regions of elevated HDV prevalence^1,13^.

The 1.7 kb RNA genome of HDV is highly variable^14,15^; phylogenetic analyses have described eight genotypes which differ by up to 30%. The greatest variety of genotypes and subtypes has been found in hyperendemic regions of western and central Africa, where genotypes 1, 5, 6, 7, and 8 are present and may be associated with better clinical outcomes than strains found elsewhere^16–19^. The influence of HDV genotype on the clinical course of infection has not been fully established, albeit there is evidence that genotype 3 viruses may cause more aggressive disease^20^ and respond better to treatment^21^. The extreme genetic diversity of HDV has impacted viral detection by molecular methods, with detection of divergent African and South American isolates proving particularly problematic^22^. Despite the global burden of HDV, only 399 genome sequences are available in the National Center for Biotechnology (NCBI) repository, while 10,550 complete HBV genomes are available in the database (accession date March 21^st^, 2018). Investigation of the genetic diversity of this highly variable virus is essential for HDV epidemiology and for the design of robust detection assays that can accommodate viral diversity regardless of the geographic region where an infection was acquired.

**Figure 1:**
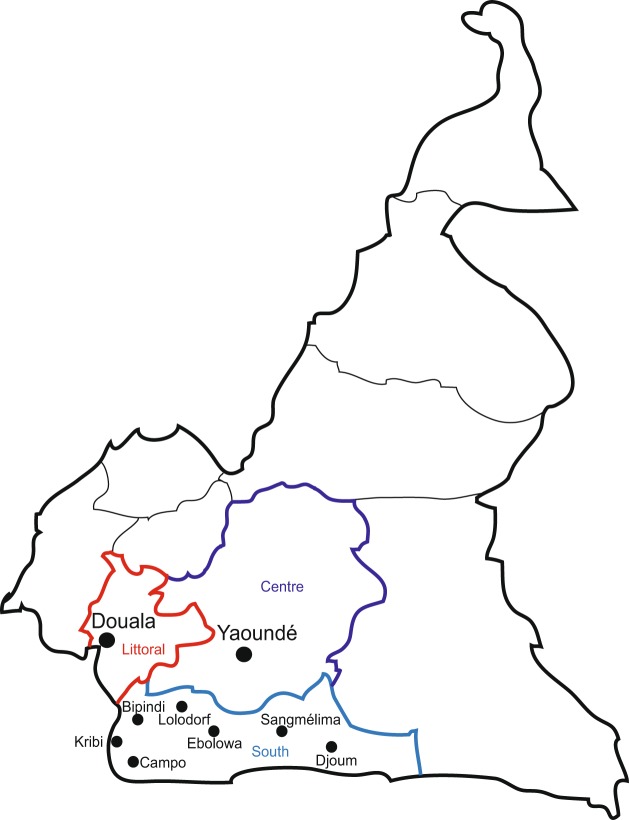
Map of study locations in Cameroon. The locations of study participants from two HIV surveillance studies included in the retrospective cohort for HDV testing are indicated on a map of Cameroon. The first study included specimens from the two largest cities in Cameroon, Douala (Littoral region, red) and Yaoundé (Centre region, violet) as indicated by large black dots. The second study included specimens from 14 towns in the South region (blue), with major study sites indicated by small black dots.

We recently completed a large scale surveillance study in the South region of Cameroon (Fig. 1)^23^. Amongst the 13,700 participants in the study, HBV prevalence was 10.45% from 2011–2015,^23^ although the HDV prevalence was unknown. In parallel, samples have also been collected in an ongoing HIV diversity surveillance study in urban centers of Cameroon (Fig. 1), although HBV or HDV prevalence has not previously been examined in this cohort^24^. Herein, we retrospectively interrogated the HDV status of 1928 HBsAg-positive plasma samples from these two surveillance studies using recently developed automated research assays for detection of HDV IgG and HDV RNA on the Abbott ARCHITECT and m2000 platforms, respectively^25^, and found a high prevalence of both HDV exposure (46.73% seropositive) and active infection (34.2% HDV RNA positive). Furthermore, we report sequence data from 211 patient samples (145 near complete genomes and 66 partial sequences), with genotypes 1, 6, 7, and 8 present in the study population. Overall, the high prevalence and diversity of HDV infection observed within this population highlights the importance of HDV testing in the care of HBV infected individuals in Cameroon.

## Materials and Methods

### Samples

The retrospective cohort included HBsAg-positive specimens from two surveillance studies conducted in Cameroon from 2010–2016^23,24^. Both studies were approved by the Cameroon National Ethical Review Board, the Faculty of Medicine and Biomedical Science IRB, and the Ministry of Health of Cameroon^23,24^. In accordance with study protocols, written informed consent was provided and plasma was collected anonymously. Unique populations were sampled in the two studies included in this retrospective cohort: participants were recruited from blood bank donors, hospitals, and chest clinics in the urban centers of Douala and Yaoundé^24^, or patients with illness of unknown etiology, antenatal patients, and participants of door-to- door and voluntary testing campaigns were recruited from 14 villages and towns in the South region of Cameroon^23^. All samples were tested on-site in Cameroon for HIV according to the national algorithm using the Determine HIV1/2 test (Alere International Limited, Zug, Switzerland) and/or Murex HIV Ag/Ab Combo test (DiaSorin, Saluggia, Italy). All specimens in the combined retrospective cohort were pre-screened using the Abbott ARCHITECT HBsAg (1L80) or HBsAg Qualitative (4P53) assays (Abbott Diagnostics, Abbott Park, IL) and confirmed by secondary screening for HBsAg by a previously described research mutant screening assay^23^. A limited number of samples were also tested for HBV viral loads using the Abbott HBV real time assay (2G34, Abbott Molecular, Des Plaines, Illinois, USA). HBV sequencing was performed on a subset of samples as previously described^23^. All samples were tested for HIV using ARCHITECT assays (2P37 or 3A77, Abbott Diagnostics, Abbott Park, Illinois, USA).

#### HDV testing algorithm

Initial HDV IgG version 1.0 testing was performed on HBsAg reactive samples collected in 2010–2015 followed by reflex screening of serology reactive samples for HDV RNA. To correct for potential deficiencies of IgG only screening, HDV seronegative samples were mini-pooled (5 per pool) and tested for HDV RNA. HDV viremic samples without evidence of HDV IgG were subjected to additional diagnostic testing for IgM or a modified version of the IgG assay using a recombinant HDV large antigen (version 1.5). The additional diagnostic testing presented deficiencies in the original HDV IgG assay using peptides and a subsequent version 2.0 assay was developed. The version 2.0 HDV IgG assay was applied to screen all specimens collected in 2016. In parallel, this set of specimens was screened for HDV RNA regardless of serology results. A flowchart detailing the complete testing algorithm can be found in Figure S1.

### HDV antibody testing

#### HDV IgG version 1.0

The version 1.0 research use prototype assay for the detection of HDV IgG on the ARCHITECT immunoassay platform has demonstrated 100% specificity, 100% sensitivity, and 69.6% positive predictive value for HDV RNA in a seropositive sample as previously described^25^ (Abbott Diagnostics, Abbott Park, IL, USA). A provisional cut-off for the assay was previously established based on screening an HBsAg and HBV DNA negative population from US volunteer donors (non-endemic area) (ProMedDx, LLC, Norton, MA was set at median plus 10 standard deviations (SD) to result in 100% specificity^25^.

#### Additional serologic testing

The HDAg protein (R18420, Meridian, Memphis, Tennessee, USA) was coated onto magnetic microparticles and used in an indirect IgG assay format as described above. A signal to noise (S/N) >/= 10.0 was considered positive. HDV IgM testing was performed by modifying the HDV IgG assay using peptides by replacement of the conjugate with an acridinium conjugated mouse anti-human IgM antibody (Abbott Diagnostics, Abbott Park, Illinois, USA). A signal to noise (S/N) >/= 10.0 was considered positive.

#### HDV IgG version 2.0

A refined HDV IgG assay was designed to contain both HDV peptides and recombinant antigen. Specificity was established using a US negative donor population and provisional cut-off was determined.

### HDV Molecular testing

HDV RNA was detected as described previously^25^. Quantitation was performed using the World Health Organization (WHO) HDV nucleic acid test standard (Paul Ehrlich Institute, Germany). Where sample volume was limiting, samples were diluted 1:10 or 1:100 in HBV negative normal human plasma prior to extraction; final quantitation was adjusted to account for dilution. Seronegative specimens collected in 2010–2015 were screened for HDV RNA in pools of 5 followed by dissection of positive pools. All other specimens were tested individually as indicated in Figure S1.

### Sanger sequencing and phylogenetic analyses

Remaining volumes of HDV RNA eluates from specimens with viral load >4.5 log IU/ml were used as templates for sequencing amplicons covering 3 overlapping regions of the genome as previously described^25^. Sanger sequencing of PCR products was completed as previously described^23^. Resulting sequences were analyzed and classified by phylogenetic analysis as previously described^25^. Divergent sequences branching basal to known references were analyzed to identify recombinants using the SimPlot v3.5.1 software^26^.

### Data availability

The complete and partial HDV genome sequences were deposited in Genbank (MG711661 - MG711805).

### Statistical analysis

Confidence intervals were determined by the Wilson Score method^27^ in Microsoft Excel 2016.

## Results

### Study population

Blood samples collected from participants of two viral surveillance studies conducted from 2010–2016 were screened to identify N = 1928 HBsAg-positive specimens with sufficient volume for HDV screening^23,24^. Of these HBsAg positive specimens, 79.6% were obtained from participants in towns in South Cameroon through a large-scale HIV/HBV/HTLV surveillance study^23^; whereas 20.4% were collected from participants in the two largest cities in Cameroon, Douala and Yaoundé, through an HIV diversity surveillance study^24^. Specimen collection sites were blood banks, hospitals, clinics, and voluntary testing campaign locations in the Littoral, Centre, and South regions of Cameroon as shown in Fig. 1. Demographic data collected from N = 1702 participants indicated that 52% (896/1702) of were female, with ages ranging from 2 to 91 and an average age of 31 years old (Table 1). Previous serological screening for HIV indicated that 48.9% (942/1928) of the study population was HIV positive (Table 1). All N = 1928 specimens were screened for HDV IgG antibodies and N = 1850 of these specimens were screened for HDV RNA by the testing algorithm shown in Figure S1, with supplemental testing for IgM and revised versions of the HDV IgG assay for discordant specimens as indicated.

**Table 1:** Demographic and overall prevalence summary.

	ALL SAMPLES		HDV SEROPOSITIVE		HDV SERONEGATIVE	
TOTAL	1928		901	46.7%	1027	53.3%
MEAN AGE	31.4		29.9		32.6	
AGE RANGE	(2–91)		(2–64)		(5–91)	
MALE	806	41.8%	352	39.1%	454	44.2%
FEMALE	896	46.5%	385	42.7%	511	49.8%
SEX UNREPORTED	226	11.7%	164	18.2%	62	6.0%
HIV POSITIVE	942	48.9%	390	43.3%	552	53.75%
HIV NEGATIVE	986	51.1%	511	56.7%	475	46.25%

Percentages are calculated for the total number of samples indicated in each column category.

### HDV prevalence

**Figure 2:**
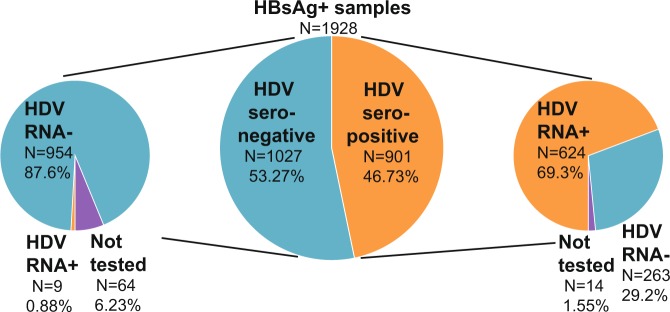
Prevalence of anti-HDV antibodies and HDV RNA. Pie charts summarizing results of HDV serological testing (center) and HDV RNA testing for HDV IgG or IgM seropositive samples (right) and HDV seronegative samples (left) for all HBsAg+ samples in the cohort.

Overall, serological testing revealed a high prevalence of exposure to HDV: 901/1928, 46.73% [95% CI; 44.51–48.96%] samples were reactive for HDV IgG or IgM antibodies amongst HBsAg positive carriers from Cameroon (Table 1, Fig. 2*)*. Molecular screening identified HDV RNA in N = 633 specimens (34.2%), with an average viral load of 4.53 (±1.9 standard deviation) log IU/ml and a maximum viral load of 9.77 log IU/ml. Within the HDV seropositive cohort; 69.25% (624/901) of samples were also positive for HDV RNA (Fig. 2). Nine HDV RNA positive seronegative samples had HDV viral loads that were less than 1.5 log IU/ml, with only 2 exceeding 100 IU/ml. These specimens were collected from 4 different cities and towns, including Yaounde, Sangmelima, Kribi, and Ebolowa, indicating that RNA positive, seronegative results were not unique to one particular site. Notably, two of the HDV RNA positive, seronegative specimens were from HIV positive donors. The overall seroprevalence of HDV in the HIV positive cohort was 41.4% (390/942), and HDV RNA was present in 26.5% (250/942) of all HIV positive specimens (Table 1). In contrast, HDV prevalence was higher in the HIV negative study cohort, with HDV seroprevalence being 51.8% (511/986) and HDV RNA prevalence being 38.8% (383/986) amongst HIV negative participants.

**Figure 3:**
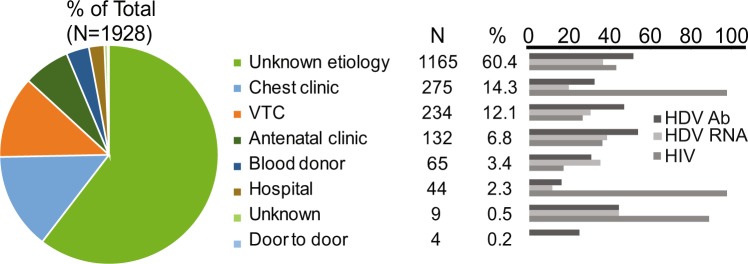
Summary of specimen collection source types. At right is a pie chart showing representation of collection risk group categories among the 1928 samples tested within this study. The N and % per collection type is given at center. Percent HDV serology or molecular testing, as well as HIV positive status, by collection category is given in the bar graph (right) with % positive results given relative to the number of all tested samples within respective sample population. VTC, volunteer testing campaign.

**Figure 4:**
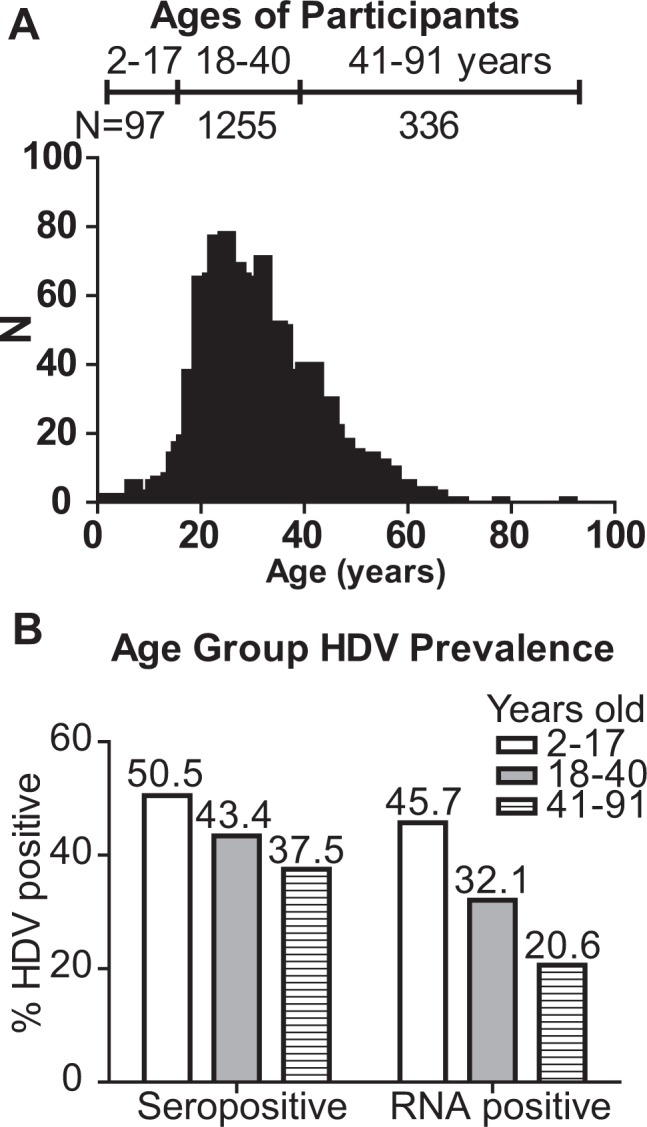
Testing results by patient age. (**A**) Distribution of patient ages in the study population. (**B**) HDV seroprevalence and RNA prevalence by age group. The number of participants per age group is noted in A.

The prevalence of HIV in the study population (48.86%) was much higher than the observed HIV prevalence in the general population of Cameroon in a 2011 national survey (7.2%)^28^, and is likely due to the bias of the study sampling towards patients seeking healthcare. The majority of samples in the study were obtained through collection of specimens from patients with illness of unknown etiology (60.4%) and other hospital settings (e.g. Chest clinic, antenatal, hospital patient) (Fig. 3). The age range of HBsAg-positive samples in this study was 2–91 years (Table 1), with the highest number of participants being within the ages of 18–40 and a mean age of 31 (Fig. 4a). We observed a downward trend in HDV seropositive and RNA positive rates with increasing ages of participants (Fig. 4b). No appreciable gender difference in HDV prevalence was observed (Table 1).

### HDV phylogenetic analyses

**Figure 5:**
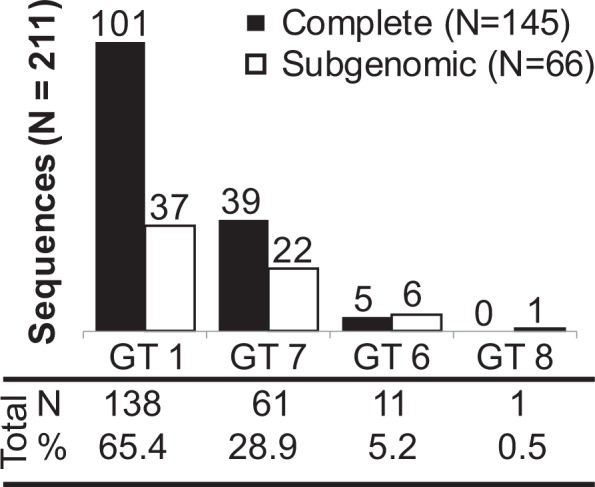
Summary of HDV genotypes observed in the study cohort. Distribution of complete/near complete and subgenomic sequences by genotype are given.

**Figure 6:**
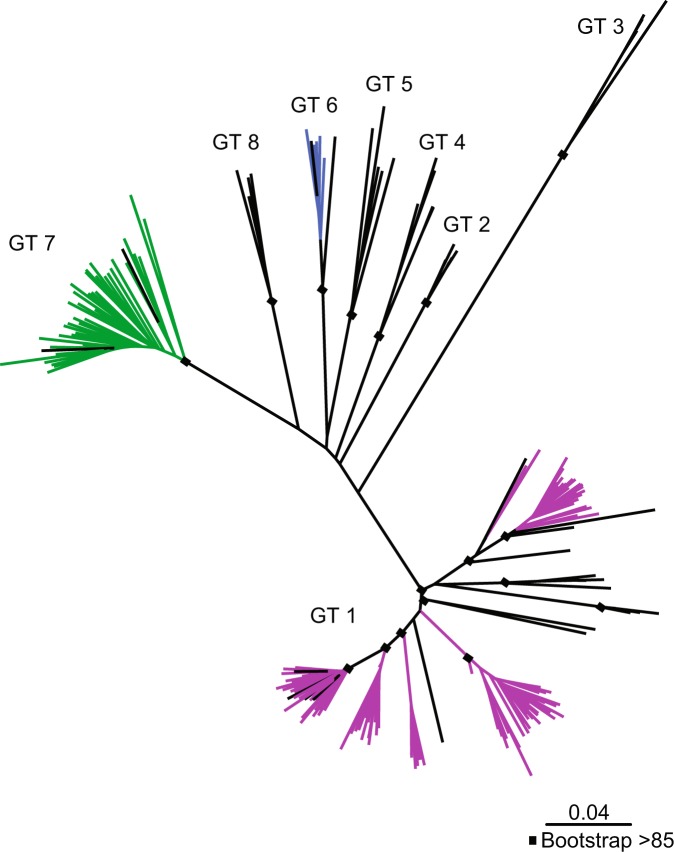
HDV genomic phylogenetic tree of complete and near complete genomes. The phylogenetic tree for N = 145 near complete genomes and N = 43 reference strains is presented with references depicted in black and relevant nodes with bootstrap values >70 labeled with a black box. Genotypes for each major branch are labeled (GT1-8).

To determine the genetic diversity of HDV in the study population, molecular characterization and phylogenetic analysis was performed for specimens with viral loads >4.5 log IU/ml. Sequences were obtained from N = 211 specimens that were RT-PCR positive in at least one of three overlapping regions targeted. In total, 145 complete or near complete genomes of at least 1 kb in length and 66 shorter sequences were aligned to reference sequences of the same length and classified according to branching patterns in neighbor joining trees. Amongst all of the HDV sequences generated, genotypes 1, 6, 7, and 8 were identified in the study population, indicating a high level of HDV diversity is present in Cameroon (Figs 5 and 6). Genotype 1 was predominant (65.4%, 138/211), with three sample clusters branching distinct from reference sequences (bootstrap values > 85) (Fig. 6). Genotype 7 was also well-represented (28.9%, 61/211), including five sequences branching basal to genotype 7 reference sequences with high bootstrap values (>85) (Fig. 6). Genotypes 6 (5.2%, 11/211) and 8 (0.5%, 1/211) were less common. Sequences branching basal to references were examined in SimPlot for potential recombinant breakpoints, though no evidence of recombination was observed.

HBV genotype data was available for 334 samples in this cohort^23^; 32% of HBV genotyped samples were genotype A, 67.4% were genotype E, with 2 samples having dual infections of genotype A and E^23^. HDV seroprevalence was highest in the HBV genotype E cohort (Figure S2), consistent with a higher overall prevalence of genotype E. Among the 25 samples with both HBV and HDV sequence data, a clear co-circulation pattern was not observed: HDV genotype 1 (2A, 11E), HDV genotype 6 (1A, 1E), HDV genotype 7 (2A, 8E).

## Discussion

We investigated HDV seroprevalence and RNA prevalence in a retrospective cohort of 1928 HBsAg-positive specimens collected from 2010–2016 in Cameroon. Within these samples, we report a high prevalence of HDV infection: the observed overall seroprevalence was 46.73%, indicating a high rate of HDV exposure. Further, HDV RNA testing revealed that 34.2% of patients were infected with actively replicating HDV at the time of sample collection. In Cameroon, the reported HDV seroprevalence ranges from 7 to 62% depending on the population tested and the assay used for screening^13,16,18,29–31^. When HDV status is stratified by age, a downward trend is observed wherein the prevalence of HDV is reduced with increasing age in this cohort (Fig. 4). This trend was evident to a lesser degree with HDV seroprevalence, likely due to sustained HDV IgG response from recovered illnesses in the older populations (Fig. 4b). Overall, our data highlight the necessity for HDV monitoring in HBV carriers of all ages within this region and support the need for HBV vaccination at an early age.

Limitations of this study include the disparate composition of the convenience population tested. We considered that the relatively high HDV prevalence observed herein could be influenced by over-representation of HIV infected individuals as 48.86% samples were HIV positive due to sample collection bias. Serological evidence of HDV infection was found in 41.4% of HIV carriers tested, which is indeed elevated compared to other studies in Cameroon^32^; however, higher HDV seroprevalence (51.8%) was observed in HIV negative samples, repudiating bias due to HIV-positive status (Table 1). Sample collection from patients seeking healthcare at clinical sites could also introduce bias toward elevated HDV prevalence, though we note that HDV prevalence was not higher in samples collected from clinical sites (unknown illness, chest clinic, hospital) compared to healthy donors (voluntary testing campaign, blood donors) (Fig. 3).

Another study complication was the use of prototype assays. The initial HDV IgG test (version 1.0) detected 691/1528 HBsAg positive samples with 501/691 having active HDV infection. By screening seronegative samples for HDV RNA, we identified an additional 34 samples with detectable HDV RNA. Using additional serology testing (for IgM or using recombinant antigen for IgG testing) we found evidence of HDV antibodies in the majority (26/34) of these initially seronegative samples, which included genotype 1, 6, and 7 specimens (Table S1). The supplemental testing informed revision of the anti-HDV assay (version 2.0) (Figure S1). Overall, using anti-IgG as a primary screen resulted in 9 seronegative/RNA positive and 3 IgM only/RNA positive samples, suggesting the value of IgM testing is limited in highly endemic areas. There were nine HDV RNA positive samples that were identified with no evidence of HDV seroconversion (Table S1), leaving the possibilities that these specimens were from acute infections, had other unknown underlying clinical complications, or evaded detection with the current prototype anti-HDV assays. All had HDV viral loads less than log 1.5 IU/ml and could not be sequenced (Table S1, indicated in bold). Unfortunately, no additional clinical data was available for these samples beyond the diagnostic testing we could perform. Interestingly of these 9 samples, 4 were from blood donors in the same collection site and year (Yaoundé, 2015). Overall, HDV seroprevalence was higher than RNA prevalence, with the exception of the blood donor cohort (Fig. 3). Little is known about the diagnostic profile of incident HDV infections since most HDV surveillance reports primarily rely on serology testing^18,33–36^. The seroconversion profile of a chimpanzee superinfected with HDV indicates that RNA is detectable in the absence of IgG or IgM for 30 days^25^; acute infections are likely HDV RNA positive prior to seroconversion in humans as well. Indeed, human HDV IgM and IgG responses have been reported to be late or undetected during HDV infection^37–39^. Future HDV surveillance studies may find additional RNA positive specimens if seronegative samples are screened, potentially allowing for better estimations of the prevalence of incident HDV infections. To our knowledge, HDV incidence has not previously been measured in a surveillance study, although it can be inferred from HDV prevalence rates amongst HBV incident infections as previously demonstrated in an Italian cohort^40^.

In addition to HDV molecular and serologic testing, we sequenced 211 HDV RNA positive samples, generating complete or near complete genomes from 145 samples and 66 sub-genomic sequences. Phylogenetic analyses of sequence data revealed genotype 1 to be dominant with additional sequences classified to genotype 7, genotype 6, and genotype 8, consistent with other analyses from central Africa^17,18,33^. Evidence of genotype 1 sub-clades has been noted in Africa^15,18,33^ and elsewhere^41,42^; the sequences obtained in the present study provide further evidence of rich diversity within this clade and, overall, expand greatly upon available HDV sequence data. Specifically, we find 3 branches of 13, 7, and 32 genotype 1 sequences, respectively, with bootstraps of >85 that cluster independent of reference sequences. Likewise, we find evidence of further genetic diversity in genotype 7 in our phylogenetic analysis: five Cameroon sequences are basal to reference samples. No evidence of recombination was observed in any of these sequences. Of note, no representatives of genotype 5 were observed in this cohort despite this genotype being previously identified in the region^18^. Further, we identified only one subgenomic sequence representative of genotype 8 within this cohort, despite evidence that this genotype is circulating within central and western Africa^17,36,43^.

Overall, we report high prevalence of HDV in Cameroon, including diverse viruses from genotypes 1, 6, 7, and 8; divergent sequences reported here increase the known diversity of these clades. Our data indicate that HBV patients in Cameroon are at elevated risk for severe hepatitis and death due to illness compounded by HDV infection. HDV worsens hepatitis symptoms and clinical outcomes, including a higher likelihood of progression to hepatocellular carcinoma^30^. Screening and diagnosis of HDV in HBV patients in Cameroon might identify individuals at increased risk for developing liver disease. Due to the high endemic seroprevalence of HDV in HBsAg carriers in Cameroon, access to serologic testing from dried blood spots could provide a means to diagnose HDV in high risk populations with limited access to venipuncture, although studies comparing assay sensitivity and specificity for dried blood spot specimens are necessary to evaluate this diagnostic approach. Importantly, HBV vaccination could help reduce the hepatitis burden in Cameroon. As HDV continues to diversify, HDV surveillance will be critical for monitoring emerging strains of HDV that may challenge diagnostic tests.

## Electronic supplementary material

Supplementary information
