# Supplementary material for: High prevalence of hepatitis delta virus in Cameroon
Source: Sci Rep. 2018 Aug 2;8:11617. doi: 10.1038/s41598-018-30078-5 (PMC6072717; doi:10.1038/s41598-018-30078-5)
Supplement: Supplementary file 1 — Supplementary information [file 41598_2018_30078_MOESM1_ESM.pdf]

Supplementary Information for:

High prevalence of hepatitis delta virus in Cameroon

Emily K Butler[1+], Mary A Rodgers[1+*], Kelly E Coller[1], Devin Barnaby[2], Elizabeth Krilich[2], Ana Olivo[1], Michael Cassidy[1], Dora Mbanya[3], Lazare Kaptue[4], Nicaise Ndembi[5], Gavin Cloherty[1]

[+] These authors contributed equally

Affiliations:

1 Abbott Laboratories, Abbott Park, IL, USA

2 Franciscan Institute for Science and Health, Franciscan University of Steubenville, Steubenville, OH, USA

3 Université de Yaoundé I, Yaoundé, Cameroon

4 Université des Montagnes, Bangangté, Cameroon

5 Institute of Human Virology, Abuja, Nigeria

Corresponding Author: Mary A Rodgers, 100 Abbott Park Rd, Abbott Park, IL 60064, mary.rodgers@abbott.com, Phone: 224-668-8936, Fax: 224-667-1401

Figure S1

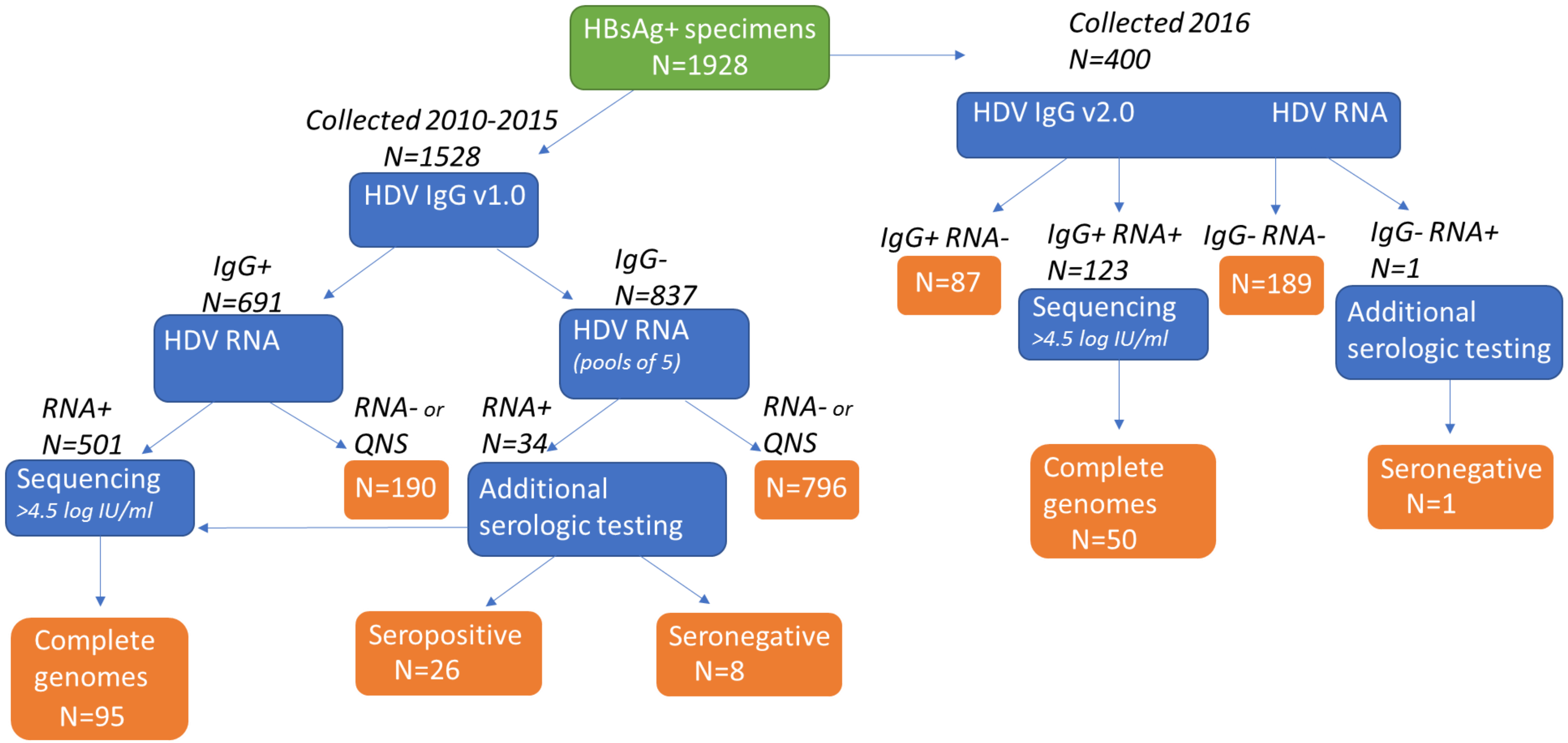

Figure S1. Testing workflow: Testing algorithms for specimens collected in 2010-2015 (left) and 2016 (right) are shown. For the 2010-2015 cohort, a total of 34 HDV RNA positive seronegative samples were identified. With additional serologic testing, 26 of these 34 were determined to be HDV seropositive: 3 samples were positive for HDV IgM only and 23 samples had IgG antibodies detectable using recombinant HDAg. Eight of the 34 seronegative samples were HDV RNA positive with no evidence of HDV antibodies (IgM or IgG) in the 2010-2015 cohort. Given the improvements in serodetection by testing with the recombinant HDAg, a revised assay incorporating both HDAg peptides and recombinant protein (version 2.0) was used to screen and additional set of HBsAg samples collected in 2016, with parallel testing for HDV RNA. One RNA+ seronegative specimen was identified in the 2016 cohort with viral load of 1.14 log IU/ml, additional serologic testing determined there was no evidence of HDV antibodies (IgM or IgG) in this specimen. *Quantity not sufficient (QNS)*

Table S1

Sample	Year	Donor risk group	Site	HIV status	HDV RNA: HDV VL (log IU/ml)	HDV RNA: HDV GT	HDV IgG S/CO	HDV supplemental: HDV IgM S/N[a]	HDV supplemental: HDV IgG (rAg) S/N[a]
**Evidence of HDV IgM antibodies only**									
1	2013	Unknown illness	Kribi	NEG	0.44	VLNS	0.02	34.45	1.33
2	2011	VTC	Kribi	NEG	1.63	VLNS	0.08	12.08	QNS
3	2013	Unknown illness	Ngovagang	POS	8.05	1	0.17	39.01	QNS
**Evidence of HDV IgG**									
4	2013	Blood donor	Yaounde	QNS	1.82	VLNS	0.28	100.47	77.26
5	2013	Unknown illness	Djoum	NEG	4.69	1	0.98	31.89	81.64
6	2013	Unknown illness	Sangmelima	POS	5.27	1	0.48	*8.01*	87.18
7	2014	Unknown illness	Bipindi	POS	4.04	1	0.86	3.32	86.57
8	2013	Unknown illness	Ebolowa	NEG	6.78	6	0.92	1.30	77.61
9	2010	Chest clinic	Yaounde	POS	3.82	VLNS	0.78	1.02	84.23
10	2015	Antenatal clinic	Ebolowa	POS	1.94	VLNS	0.72	1.79	10.47
11	2014	Unknown illness	Kribi	NEG	5.26	1	0.52	3.16	37.97
12	2015	Antenatal clinic	Ebolowa	POS	4.12	QNS	0.41	0.89	49.20
13	2014	Unknown illness	Lolodorf	NEG	6.07	1	0.19	1.86	*8.60*
14	2011	VTC	Sangmelima	NEG	7.31	7	0.52	4.79	30.60
15	2013	Unknown illness	Ebolowa	POS	8.41	1	0.66	0.51	39.70
16	2015	VTC	Sangmelima	NEG	5.82	1	0.57	1.02	44.00
17	2015	Blood donor	Yaounde	NEG	0.58	VLNS	0.06	0.83	*9.27*
18	2014	Unknown illness	Kribi	POS	0.66	VLNS	0.29	1.14	92.10
19	2015	Chest clinic	Yaounde	POS	2.64	VLNS	0.42	3.37	83.77
20	2012	Unknown illness	Ngovagang	NEG	3.18	7	0.12	3.25	91.98
21	2011	Antenatal clinic	Ebolowa	NEG	2.94	VLNS	0.74	2.39	100.59
22	2013	Chest clinic	Yaounde	QNS	5.33	1	0.10	0.54	50.81
23	2010	Chest clinic	Yaounde	POS	5.78	1	0.08	2.10	98.89
24	2011	VTC	Kribi	NEG	6.99	7	0.17	0.85	98.89
25	2012	Hospital patient	Yaounde	POS	6.75	1	0.06	1.06	*6.60*
26	2011	Blood donor	Yaounde	POS	3.46	7	0.08	*6.92*	26.80
**No evidence of HDV seroconversion**									
27	2013	Unknown illness	Ebolowa	NEG	0.48	VLNS	0.02	3.29	1.58
28	2013	Unknown illness	Kribi	NEG	1.55	VLNS	0.01	4.98	1.06
29	**2015**	**Blood donor**	**Yaounde**	NEG	0.75 [b]	VLNS	0.35	0.85	5.48
30	**2015**	**Blood donor**	**Yaounde**	NEG	0.79 [b]	VLNS	0.18	0.55	0.61
31	**2015**	**Blood donor**	**Yaounde**	NEG	0.81 [b]	VLNS	0.02	1.42	0.58
32	**2015**	**Blood donor**	**Yaounde**	NEG	1.23 [b]	VLNS	0.05	0.84	0.16
33	2013	Chest clinic	Yaounde	QNS	2.25	VLNS	0.10	1.42	0.83
34	2014	Unknown	unknown	POS	3.75	1	0.04	0.44	0.90

[a] Shown are signal to noise values, S/N>10 considered as evidence of HDV antibodies. Original cut-off in peptide IgG assay was 35*NC. Values >6 are considered elevated, indicated in italics.

[b] Not determined due to insufficient volume, tested with recombinant HDAg in IgM assay.

QNS: quantity not sufficient

VLNS: viral load not sufficient

Four seronegative blood donor specimens with viral load below <1.5 mentioned in the text are indicated in bold

Figure S2

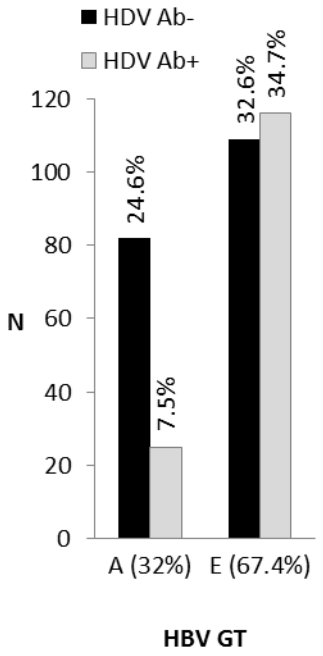

Figure S1. HBV genotype data. HBV genotype data was available for 334 samples in the study cohort; 2 HBV GT AE samples were HDV negative. Results for HBV GT A and E are given above by HDV seroprevalence.
